# Universal First-Trimester Screening Biomarkers for Diagnosis of Preeclampsia and Placenta Accreta Spectrum

**DOI:** 10.3390/biom15020228

**Published:** 2025-02-04

**Authors:** Angelika V. Timofeeva, Ivan S. Fedorov, Alla M. Tarasova, Yuliya V. Sukhova, Vyacheslav G. Kolod’ko, Tatiana Yu. Ivanets, Gennady T. Sukhikh

**Affiliations:** National Medical Research Center for Obstetrics, Gynecology and Perinatology Named After Academician Kulakov V.I., Moscow 117997, Russia

**Keywords:** piRNA, placenta accreta spectrum, preeclampsia, blood serum, first-trimester screening, real-time PCR, ELISA, extracellular vesicles, Clusterin

## Abstract

Background: Disruptions in epigenetic mechanisms regulating placentation, particularly imbalances in the levels of small non-coding RNAs, contribute to various pregnancy complications, including preeclampsia (PE) and placenta accreta spectrum (PAS). Given that abnormal trophoblast differentiation, invasiveness, and angiogenesis—reduced in PE and excessive in PAS—are central to the pathogenesis of these conditions, this study aimed to identify universal circulating piRNAs and their targets. Methods: Small RNA deep sequencing, quantitative reverse transcription combined with real-time polymerase chain reaction, magnetic bead-based multiplex immunoassay, ELISA, and Western blotting were employed to quantify circulating piRNAs and proteins in the blood serum of pregnant women during the 11th–14th weeks of gestation. Results: Statistically significant negative correlations were identified between PE- and PAS-associated piRNAs (hsa_piR_019122, hsa_piR_020497, hsa_piR_019949, and piR_019675) and several molecules, including Endoglin, IL-18, VEGF-A, VEGF-C, Angiopoietin-2, sFASL, HB-EGF, TGFα, and Clusterin. These molecules are involved in processes such as angiogenesis, inflammation, the epithelial–mesenchymal transition, cell proliferation, adhesion, and apoptosis. A first-trimester pregnancy screening algorithm was developed using logistic regression models based on Clusterin concentration and the levels of hsa_piR_020497, hsa_piR_019949, piR_019675, and hsa_piR_019122. Conclusions: The proposed screening tool for early pregnancy monitoring may enable the prediction of PE or PAS in the first trimester, allowing timely interventions to reduce maternal and perinatal morbidity and mortality.

## 1. Introduction

Abnormal placentation is a key pathogenetic mechanism underlying various pregnancy complications, including preeclampsia (PE), intrauterine growth restriction, and placenta accreta spectrum (PAS) [1,2]. Structural and functional abnormalities of the placenta disrupt the normal functioning of multiple maternal and fetal organ systems [3,4,5,6]. The degree of trophoblast cell invasiveness is finely regulated both during the preimplantation stage of early embryogenesis and after embryo implantation into the uterine decidua [7]. The zygote’s development to the blastocyst stage culminates in the differentiation of blastomeres into the inner cell mass, which forms the fetus, and the trophectoderm, which further differentiates into villous and extravillous trophoblast cells, shaping the placental structure. Invasion into the decidual layer of the endometrium is mediated by interstitial extravillous cytotrophoblast cells from anchoring villi, which secure placental attachment to the endometrial stroma, and by endovascular cytotrophoblast cells, which remodel uterine spiral arteries to establish appropriate blood flow. Endovascular invasion of trophoblast cells into the spiral artery’s decidual segments occurs between 8 and 10 weeks of gestation, while invasion into the myometrial segments occurs between 16 and 18 weeks [8]. In parallel with spiral artery remodeling, a multinucleated syncytiotrophoblast layer forms through the continuous proliferation and fusion of villous cytotrophoblast cells. This layer facilitates nutrient transport and gas exchange between the mother and fetus.

Decidual stromal cells of the endometrium play a critical role in regulating placentation. Under the influence of progesterone, these cells secrete interleukin-15 (IL-15), which promotes the proliferation and differentiation of natural killer (NK) cells into various subpopulations with specific gestational functions [9]. These NK cells produce angiogenic growth factors associated with spiral artery remodeling and enhance extravillous trophoblast invasion by increasing matrix metalloproteinase-9 (MMP-9) secretion up to the 18th week of gestation but subsequently secrete cytokines such as TNF-α, TGF-β, and IFN-γ, which limit excessive trophoblast invasion.

A dysregulated balance of NK cell subpopulations may lead to pregnancy complications such as PE or PAS. In PE, trophoblast cell proliferation and invasion are impaired, spiral artery remodeling is disrupted, and the placenta exhibits an anti-angiogenic phenotype [10,11]. Conversely, in PAS, the placenta exhibits an angiogenic phenotype, and extravillous trophoblast cells undergo abnormally aggressive epithelial–mesenchymal transition (EMT), resulting in high invasiveness that persists beyond the first trimester and continues throughout pregnancy [12,13].

Given the critical role of epigenetic mechanisms in regulating trophoblast differentiation, migration, and invasion [14], particularly small non-coding RNAs (sncRNAs), numerous studies have analyzed the qualitative and quantitative composition of sncRNAs in women with PE [15,16] or PAS [17,18] compared with normal pregnancies. Both these studies and our own research [19,20] have evaluated the potential of microRNAs (miRNAs) in the early diagnosis of PE and PAS.

However, no studies on the pathogenesis of PE and PAS have explored the role of another class of small non-coding RNAs, piwi-interacting RNAs (piRNAs). These RNAs not only regulate the expression of protein-coding genes but also contribute to genome stability by inhibiting transposon activity and modulating DNA methylation, thereby influencing the transcriptional activity of various genomic regions [21,22,23,24].

We were the first to reveal the role of piRNAs in determining embryo quality and implantation potential based on their expression profiles in embryo culture medium at the morula and blastocyst stages. Additionally, we identified potential piRNA target genes associated with the differentiation of blastomeres into embryoblast and trophoblast cells [25,26]. Since abnormal trophoblast cell differentiation and invasiveness—reduced in preeclampsia (PE) and excessive in placenta accreta spectrum (PAS)—are central to the pathogenesis of both pregnancy complications, this study aimed to identify universal piRNAs and their targets.

So, we hypothesized that morphofunctionally altered trophoblast and decidual cells secrete protein and piRNA molecules into maternal blood. These molecules may potentially serve as common diagnostic markers for PAS and PE but are able to differentiate two pregnancy complications from each other, in the early stages of pregnancy, such as at 11–14 weeks of gestation. This foundational research will support the development of screening tools for first-trimester pregnancy monitoring, enabling early detection of PE or PAS and facilitating timely interventions to reduce maternal and perinatal morbidity and mortality.

## 2. Materials and Methods

### 2.1. Patients

All participants were admitted to the National Medical Research Center for Obstetrics, Gynecology, and Perinatology (named after Academician V.I. Kulakov) of the Ministry of Healthcare of the Russian Federation for pregnancy and delivery management. All patients signed informed consent forms to participate, and the study was approved by the Center’s Ethics Committee. Each patient underwent comprehensive clinical and biochemical blood tests; pelvic and fetal ultrasound examinations; fetoplacental blood flow Doppler studies; cardiotocography; blood pressure monitoring; proteinuria analysis; and assessment of serum levels of PLGF, sFlt-1, PAPP-A, and β-HCG using diagnostic kits.

Based on clinical and instrumental evaluations at delivery, 41 women were divided into three groups: 15 women with normal pregnancies, 14 women with preeclampsia (PE), and 12 women with placenta accreta spectrum (PAS). Exclusion criteria included pregnancies achieved through assisted reproductive technologies, multiple pregnancies, and fetal aneuploidies. The diagnosis of PAS was confirmed through histological analysis of resected uterine wall sections showing abnormal placental invasion (accreta, increta, or percreta).

PE was diagnosed based on de novo hypertension (blood pressure ≥140/90 mmHg) after 20 weeks of gestation, accompanied by proteinuria (≥0.3 g/L per day) or evidence of acute renal failure, liver dysfunction, neurological disturbances, hemolysis, thrombocytopenia, or intrauterine growth restriction. Early-onset or late-onset PE was classified depending on whether clinical symptoms appeared before or after 34 weeks of gestation, respectively [27].

### 2.2. RNA Isolation from Blood Serum

For RNA isolation, 100 µL of cell- and debris-free blood serum (prepared by sequential centrifugation at 300× *g* for 20 min and 16,000× *g* for 10 min) was used. RNA was extracted using the “Ribo-Prep” reagent kit (Central Research Institute of Epidemiology, Rospotrebnadzor, Moscow, Russia). The protocol involved adding 300 µL of lysis buffer, incubating at 65 °C for 5 min, and precipitating the RNA by centrifuging at 12,000× *g* for 5 min. The pellet was washed twice with specialized buffers, resuspended in 25 µL of RNA buffer, and centrifuged again at 12,000× *g* for 1 min. The supernatant (20 µL) was collected and used in subsequent analyses, following the manufacturer’s instructions.

### 2.3. piRNA Deep Sequencing

cDNA libraries were synthesized using 6 µL of total RNA eluate (extracted from 200 µL of blood serum with the miRNeasy Serum/Plasma Kit) following the protocol of the NEBNext^®^ Multiplex Small RNA Library Prep Set for Illumina^®^ (Sets 1 and 2, New England Biolabs^®^, Frankfurt, Germany, cat. nos. E7300S and E7580S). Libraries were amplified for 19 cycles, purified using the QIAQuick PCR Purification Kit (Qiagen, Hilden, Germany), and subjected to 6% polyacrylamide gel electrophoresis for the isolation of 136–150 bp bands corresponding to adapter-ligated piRNAs. Sequencing was performed on the NextSeq 500 platform (Illumina, San Diego, CA, USA, cat. no. SY-415-1001). Adapters were trimmed using Cutadapt, and reads shorter than 16 bp or longer than 55 bp were filtered out. Only reads with a mean quality score above 15 were retained. The remaining reads were mapped to the GRCh38.p15 human genome and piRNABase using the Bowtie, aligner. Read counts were generated using the featureCount tool from the Subread package, with the fracOverlap option set to 0.9, ensuring at least 90% intersection with piRNA features. Differential expression analysis of piRNA counts was conducted using the DESeq2 package.

### 2.4. Reverse Transcription and Quantitative Real-Time PCR

For reverse transcription, 8.5 µL of RNA containing piRNAs (isolated from blood serum) underwent polyadenylation with poly(A) polymerase in 1× E. coli poly(A) buffer (TransgenBioTech) in a total reaction volume of 10 µL at 37 °C for 20 min, followed by incubation at 65 °C for 20 min. The resulting RNA was reverse-transcribed into cDNA in a 20 µL reaction containing 1× TS RT buffer (TransgenBioTech, Beijing, China), 0.5 mM dNTPs (Evrogen, Moscow, Russia), and 4.5 µM universal sequence-linked oligo(dT) primer (Evrogen, Moscow, Russia). The reverse transcription was carried out at 42 °C for 30 min, followed by 85 °C for 5 s. The cDNA was diluted 1:9 with RNase- and DNase-free water. For qPCR, amplification was performed using the 1× qPCRmix-HS HighROX reaction mixture (Evrogen, Moscow, Russia), with 0.2 µM of an antisense primer (specific to the universal tag sequence on the 5′ end of the cDNA) and 0.2 µM of a sense primer specific to the target piRNA (Evrogen, Moscow, Russia) on a StepOnePlus™ thermocycler (Applied Biosystems, Waltham, MA, USA). Relative piRNA expression levels were determined using the ∆Ct method, with hsa_piR_003832 (piRNAbank) as the reference RNA. The qPCR conditions were initial denaturation at 95 °C for 15 min, 40 cycles of 94 °C for 15 s, optimized annealing temperature (46.2–60 °C) for 30 s, and 70 °C for 30 s. A melt curve analysis was performed to confirm reaction specificity.

### 2.5. Protein Analysis of Blood Serum

#### 2.5.1. Magnetic Bead-Based Multiplex Immunoassay

Protein analysis was conducted on 50 µL of blood serum using the Bio-Plex Pro™ Human Cancer Biomarker Panel 2, 18-plex (cat. #171-AC600M, BioRad, Hercules, CA, USA). Capture antibodies covalently coupled to magnetic beads were used to target 18 specific proteins. After washing to remove unbound proteins, biotinylated detection antibodies were added to form sandwich complexes. These complexes were detected using streptavidin-phycoerythrin, where phycoerythrin acted as the fluorescent indicator. Fluorescence detection was performed using the MAGPIX^®^ System (Luminex Corporation, Austin, TX, USA), with results analyzed using xPONENT software, version 4.1.

#### 2.5.2. Isolation of Vesicular and Non-Vesicular Fractions for Clusterin Analysis

To isolate extracellular microvesicles, 500 µL of blood serum was processed using the miRCURY Exosome Kits (Qiagen, Germany). A 200 µL precipitation solution was added, followed by 14 h of incubation at 4 °C. The samples were centrifuged at 1500× *g* for 30 min at 20 °C. The supernatant (non-vesicular fraction) was collected in a clean tube. The pellet, containing vesicles, was resuspended in 270 µL of resuspension buffer for further analysis. The vesicular and non-vesicular fractions of blood serum were stored at −80 °C.

##### Western Blotting

Samples from the vesicular and non-vesicular fractions were diluted 100-fold in a buffer containing 50 mM Tris-HCl (Sigma-Aldrich, St. Louis, MO, USA, cat. No T4661), pH 6.8; 2% sodium dodecyl sulfate (SDS) (VWR Life Science AMRESCO, Framingham, MA, USA, cat. No Am-O227-0.1); 10% glycerol (AppliChem, Darmstadt, Germany, A4443); and 5.5% 2-mercaptoethanol (VWR Life Science AMRESCO, Framingham, MA, USA, cat. No Am-O482-0.1). The samples were denatured at 65 °C for 5 min and separated by 10% SDS-PAGE in Tris-tricine buffer (100 mM Tris, 100 mM tricine (Sigma-Aldrich, St. Louis, MO, USA, cat. No T0377), 0.1% SDS). Proteins were transferred to a PVDF membrane (0.45 µm, Immobilon, Merck Millipore Ltd., Taufkirchen, Germany, cat. No IPVH07850) using a Trans-Blot^®^ SD semi-dry transfer cell (BioRad, Hercules, CA, USA, cat. No 1703940) with two buffer systems: anode buffer (40 mM 3-cyclohexylamino-1-propanesulfonic acid (CAPS) (Sigma-Aldrich, St. Louis, MO, USA, cat. No SW18805), 60 mM Tris, pH 9.6, 15% ethanol) and cathode buffer (40 mM CAPS, 60 mM Tris, pH 9.6, 0.1% SDS). The membranes were blocked with a solution of 1% non-fat dry milk (M7409, Sigma-Aldrich); 0.1% Tween^®^ 20 (Sigma-Aldrich, USA, cat. No P1379); 50 mM Tris-HCl, pH 7.5; and 150 mM NaCl (AppliChem Panreac ITW Companies, Darmstadt, Germany, A1371). The blocked membranes were incubated with primary mouse monoclonal antibodies against the alpha subunit of clusterin (B-5, Santa Cruz Biotechnology, Dallas, TX, USA, cat. No sc-5289) diluted 1:400. This was followed by incubation with goat anti-mouse secondary polyclonal antibodies conjugated with horseradish peroxidase (R&D Systems, Minneapolis, MN, USA, cat. No HAF007) at a 1:1000 dilution. Protein bands were visualized using enhanced chemiluminescence (SuperSignal™ West Femto Maximum Sensitivity Substrate, ThermoScientific, Rockford, IL, USA, cat. No 34096) and documented with the ChemiDoc MP system (BioRad, Hercules, CA, USA, cat. No 12003154). Densitometric analysis was performed using ImageLab™ Software (version 6.0 build 25 Standard Edition, BioRad Laboratories). Protein size was determined based on the electrophoretic mobility of the PageRuler™ Plus Prestained Protein Ladder (ThermoScientific, Waltham, MA, USA, cat. No 26619). To account for transfer efficiency and image exposure differences, a reference sample from the PAS group was loaded into one well of each gel in the same quantity.

##### Enzyme-Linked Immunosorbent Assay (ELISA)

Samples from vesicular (n = 40) and non-vesicular (n = 40) fractions were diluted 2000-fold in a 1× phosphate-buffered saline (PBS) solution, pH 7.8 (BioRad, USA, cat. No 161-0780). Each well was loaded with 100 µL of the diluted sample, blank, and two-fold serially diluted standards (12.5–800 ng/mL) prepared according to the protocol of the ELISA kit for clusterin (Cloud-Clone Corp., Katy, TX, USA, cat. No SEB180Hu). Absorbance was measured using the Wallac 1420-012 Multilabel Plate Counter VICTOR3™ (PerkinElmer, Singapore).

### 2.6. Statistical Analysis

Statistical analysis was performed using scripts written in the R programming language and executed in RStudio. The normality of data distribution was assessed using the Shapiro–Wilk test. When the distribution of data was different from normal, the Mann–Whitney test for paired comparison was used. The significance threshold (*p*-value) was set at 0.05. Correlation analyses for quantitative and qualitative parameters were conducted using Spearman’s non-parametric rank-order correlation test. The 95% confidence interval for the correlation coefficient was determined using Fisher’s transformation. Logistic regression models were developed in RStudio by stepwise inclusion and exclusion of piRNA and protein markers of PAS or PE based on their contributions to the model. The diagnostic potential of the models was assessed using receiver operating characteristic (ROC) analysis, evaluating the area under the curve (AUC), statistical significance, specificity, and sensitivity.

## 3. Results

### 3.1. Profiling of piRNAs in Maternal Blood Serum at 11–14 Weeks of Gestation by Deep Sequencing and Quantitative Real-Time PCR

A retrospective study was conducted using 41 peripheral blood serum samples from pregnant women at 11–14 weeks of gestation (Table S1). The study grouped participants based on delivery outcomes: normal pregnancies (Norm), 15 women; preeclampsia (PE), 14 women (including early- and late-onset cases after 20 weeks of pregnancy); and placenta accreta spectrum (PAS), 12 women (including placenta accreta, increta, and percreta). piRNA expression profiles were obtained through deep sequencing (Table S2).

The PE group was significantly different from the Norm group in 78 piRNAs (Table S2, Sheet 1). The PAS group differed significantly from the Norm group in 38 piRNAs (Table S2, Sheet 2). A Venn diagram of the results identified 25 overlapping piRNAs between the two marker lists, 53 piRNAs were unique to PE, and 13 piRNAs were unique to PAS (Table S2, Sheet 3).

For validation using quantitative real-time PCR (qRT-PCR), the following piRNAs were selected: common markers for PE and PAS, hsa_piR_020497, hsa_piR_019949, hsa_piR_020500, hsa_piR_019825, hsa_piR_008114, and hsa_piR_020490; PE-specific markers, hsa_piR_019122, hsa_piR_016742, hsa_piR_016735, and hsa_piR_008113; and PAS-specific markers, hsa_piR_019675, hsa_piR_020829, and hsa_piR_020381. The endogenous reference RNA hsa_piR_003832 was chosen as it showed no statistically significant differences between groups (*p* = 0.93 for PE vs. Norm; *p* = 0.99 for PAS vs. Norm).

**Figure 1:**
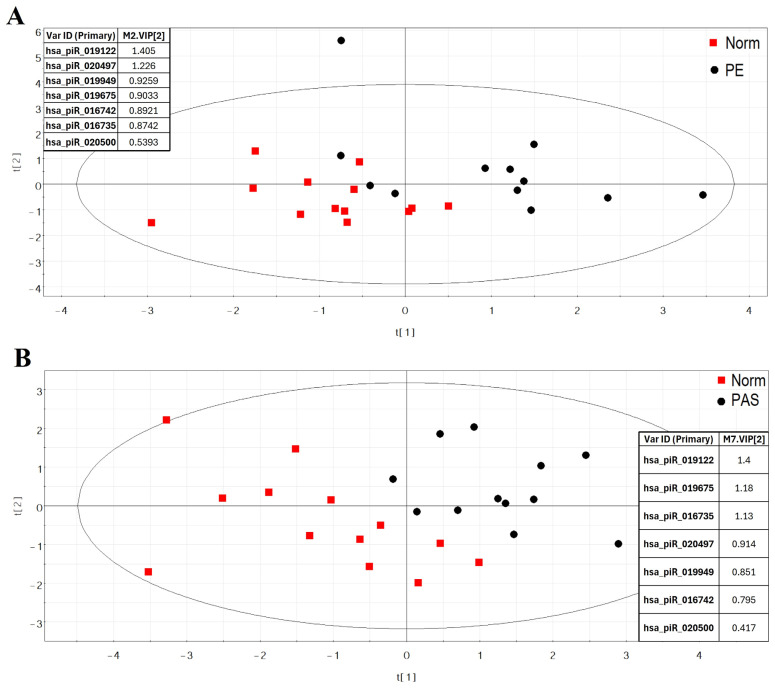
PLS-DA of RT-PCR data (-∆Ct values) for piRNAs in the blood serum of pregnant women at 11–14 weeks of gestation: (**A**) Score plot comparing Norm and PE groups. (**B**) Score plot comparing Norm and PAS groups. The VIP scores for specific piRNAs are listed to the right of the score plots.

The “-∆Ct” values for each piRNA were analyzed using partial least squares discriminant analysis (PLS-DA) (Figure 1). A distinct cluster of PE samples was observed, separate from the cluster of Norm samples (Figure 1A). The most significant contribution to this separation came from the hsa_piR_019122 and hsa_piR_020497 levels, each with a variable importance in projection (VIP) score greater than 1. PAS markers with VIP scores >1 included hsa_piR_019122, hsa_piR_019675, and hsa_piR_016735 (Figure 1B).

The clustering of samples from the three groups (Norm, PE, PAS) based on these piRNAs indicates the pathogenetic significance of these molecules in pregnancy complications in the first trimester.

To evaluate the potential use of these piRNAs as markers for detecting PE or PAS during the first-trimester screening, logistic regression was employed to construct ROC curves. Combinations of piRNAs were selected to optimize model parameters, including sensitivity, specificity, and statistical significance of the independent variables.

**Figure 2:**
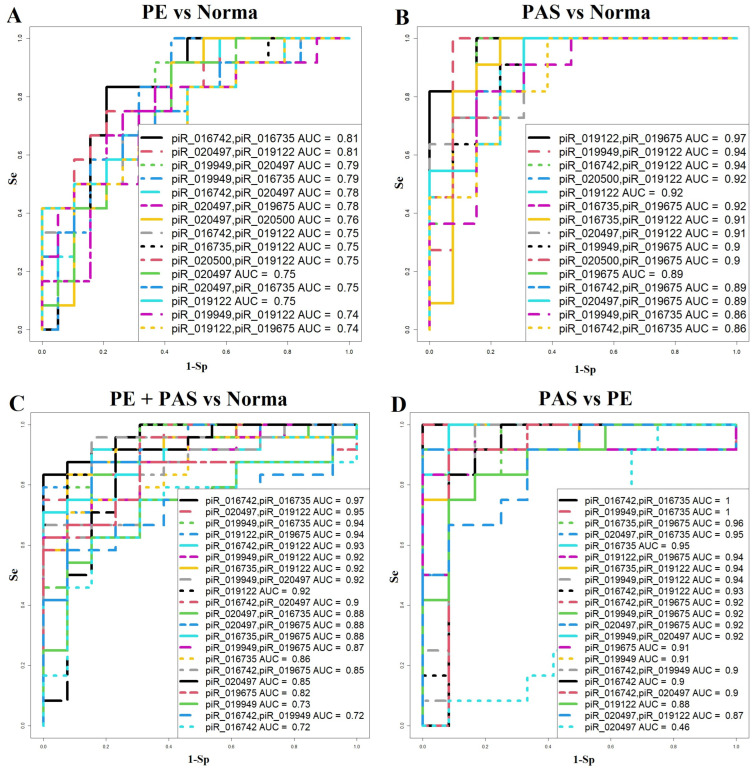
Logistic regression models for diagnosing pregnancy complications in the first trimester based on piRNA levels in maternal blood serum. (**A**) Comparison of normal pregnancy and PE groups. (**B**) Comparison of normal pregnancy and PAS groups. (**C**) Comparison of normal pregnancy and all complications (PE and PAS). (**D**) Comparison of PE and PAS groups. Sensitivity (Se) and specificity (Sp) values are indicated.

To create a specific algorithm for analyzing blood serum samples from women at 11–14 weeks of gestation, logistic regression models were developed with the diagnosis at delivery as the dependent variable (outcome):

0 = normal pregnancy; 1 = PE (Figure 2A);

0 = normal pregnancy; 1 = PAS (Figure 2B);

0 = normal pregnancy; 1 = pregnancy complications (PE, PAS) (Figure 2C);

0 = PE; 1 = PAS (Figure 2D).

The parameters of all models shown in Figure 2 are provided in Table S3. Logistic regression models with statistically significant independent variables are summarized in Table 1 and were subsequently used to create an algorithm for first-trimester screening to diagnose PE (see Section 4).

### 3.2. Protein Analysis of Blood Serum at 11–14 Weeks of Pregnancy

Multilevel diagnostic approaches are the most accurate. To create a first-trimester screening algorithm for identifying two pregnancy complications (PE and PAS), protein markers were analyzed in addition to piRNA markers.

#### 3.2.1. Protein Profiling Using Magnetic Bead-Based Multiplex Immunoassay

It is hypothesized that embryonic genome activation involves pathways associated with cancer, regulated by cancer-associated transcription factors [28]. Disruptions in epithelial–mesenchymal transition, proliferation, and differentiation of trophoblast cells into subtypes critical for invasion of the decidual endometrium and placentation underlie the development of PE or PAS.

**Figure 3:**
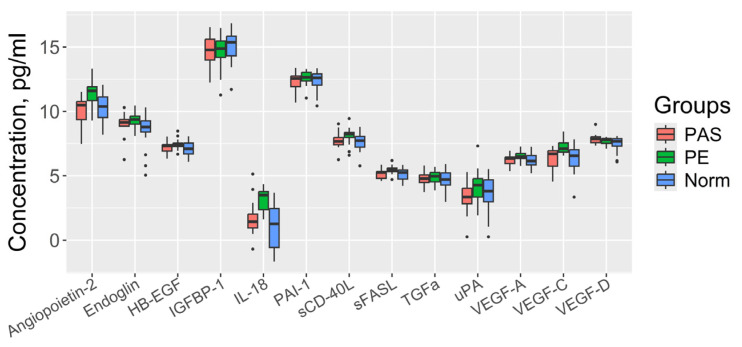
Protein analysis of maternal blood serum at 11–14 weeks of gestation using the Bio-Plex Pro human cancer biomarker assay.

To analyze the protein profiles of maternal blood serum at 11–14 weeks of gestation, the Bio-Plex Pro Human Cancer Biomarker Assay (Bio-Rad) was used. This method employs magnetic beads conjugated with antibodies for 18 soluble biomarkers involved in angiogenesis, inflammation, cell proliferation, adhesion, and apoptosis (EGF, IL-6, Endoglin, sCD-40L, sFASL, TNFa, IL-18, VEGF-D, VEGF-A, Angiopoietin-2, PLGF, IL-8, HB-EGF, PAI-1, IGFBP-1, VEGF-C, TGFa, and uPA). Figure 3 presents the results for 13 biomarkers; five proteins (EGF, IL-6, TNF-α, PLGF, and IL-8) were excluded because their concentrations exceeded the detection limits of the assay. The statistical significance of differences among groups (Norm, PE, and PAS) is detailed in Table 2.

**Figure 4:**
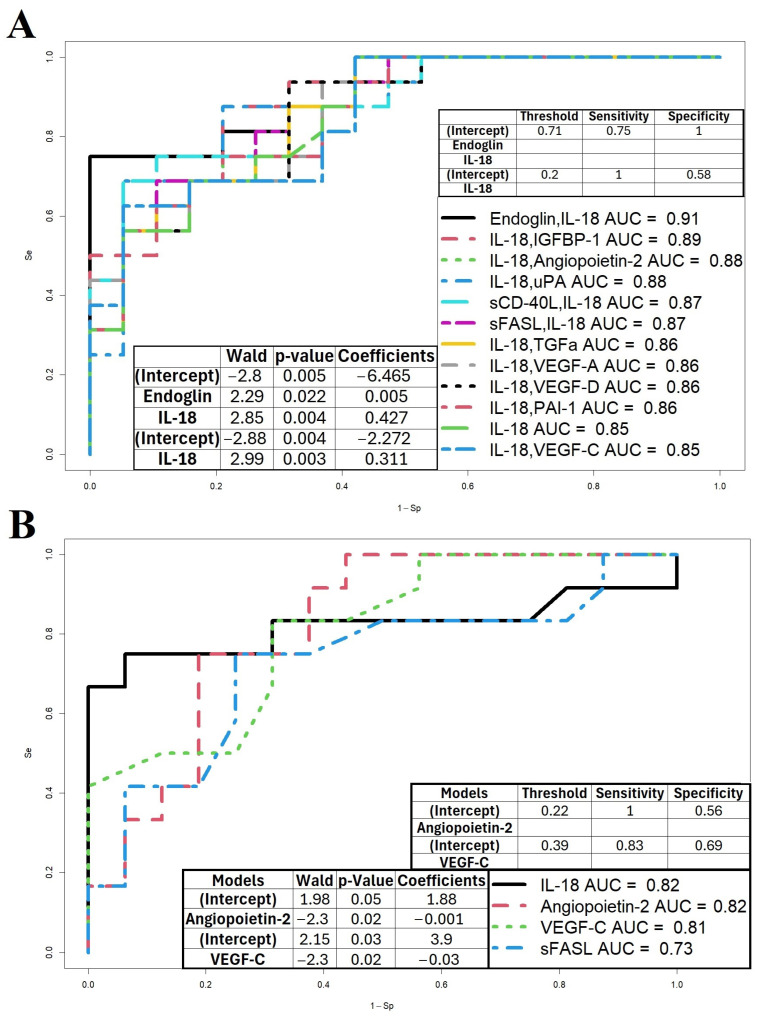
Logistic regression models for first-trimester pregnancy complication screening. (**A**) Model distinguishing Norm and PE groups. (**B**) Model distinguishing PE and PAS groups.

When comparing the PAS group with the Norm group, no significant differences in the levels of 13 proteins were found. When comparing the PE group with the Norm group and with the PAS group, statistically significant differences were found in the levels of Angiopoietin-2, IL-18, VEGF-C, sFASL, and Endoglin, which differentiated the PE group from the Norm group. To use these marker proteins in clinical practice, logistic regression models were built (Figure 4), where the dependent variable (response variable) was the diagnosis at the time of delivery: 0, physiological pregnancy; 1, PE (Figure 4A); 0, PE; and 1, PAS (Figure 4B). The parameters of the logistic regression models with statistically significant independent variables are provided in the insets of Figure 4A,B. The model in Figure 4A, based on Endoglin and IL-18 concentrations, demonstrated 100% specificity for identifying cases without PE and 75% sensitivity for identifying cases with PE. The model in Figure 4B, based on Angiopoietin-2 concentration, demonstrated 100% sensitivity for identifying PAS cases and 56% specificity for distinguishing PE cases.

Due to the absence of significant differences between the PAS and Norm groups, it was not possible to develop a unified algorithm for detecting these pregnancy complications. Thus, these proteins cannot be considered universal markers for diagnosing both PE and PAS but may serve as markers specifically for PE in first-trimester screening.

#### 3.2.2. Clusterin Analysis in Maternal Blood Serum Using Western Blotting and ELISA

Clusterin is involved in processes induced by endoplasmic reticulum stress, acting as both an intracellular and an extracellular chaperone [29,30]. Its expression in cytotrophoblast, syncytiotrophoblast, and extravillous trophoblast cells [31], as well as its role in inhibiting epithelial-mesenchymal transition (EMT) during trophoblast phenotype transformations [31], prompted an investigation of clusterin levels in various fractions of maternal blood serum (vesicular and non-vesicular). The study aimed to evaluate its potential as a universal marker for diagnosing PE or PAS in the first trimester.

**Figure 5:**
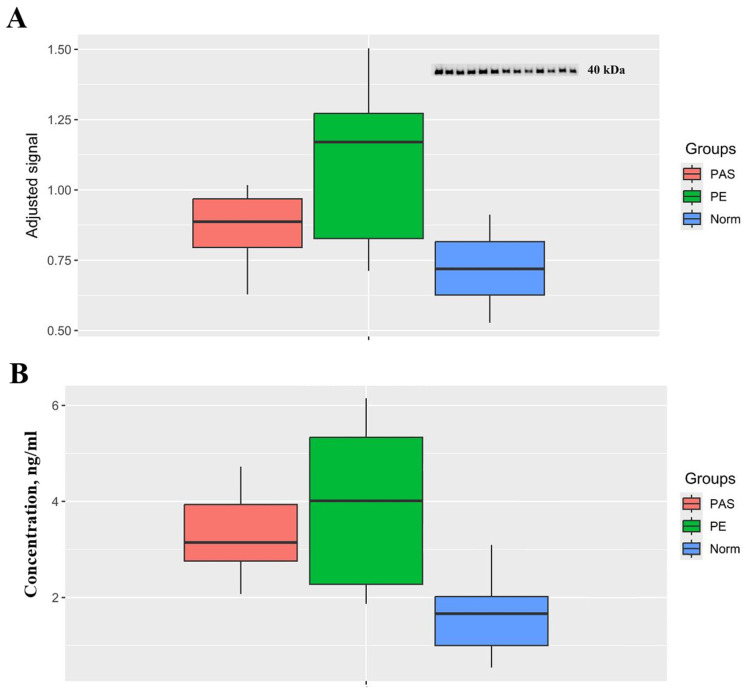
Clusterin levels in the vesicular fraction of maternal blood serum at 11–14 weeks of pregnancy. (**A**) Relative clusterin levels from Western blotting. A representative blot (top-right inset) shows chemiluminescent signal intensity at ~40 kDa, corresponding to the α-subunit of the secretory form of clusterin. (**B**) Clusterin concentration (ng/mL) measured by ELISA. Original Western blot images can be found in Supplementary File S1.

Using Western blotting and ELISA as detailed in Sections Western and Blotting and Enzyme-Linked Immunosorbent Assay (ELISA), significant differences were observed in clusterin levels between the Norm group and each of the PE and PAS groups in the vesicular fraction of blood serum at 11–14 weeks of pregnancy (Figure 5, Table 3). No significant differences between groups were identified in the non-vesicular fraction of blood serum by either method.

**Figure 6:**
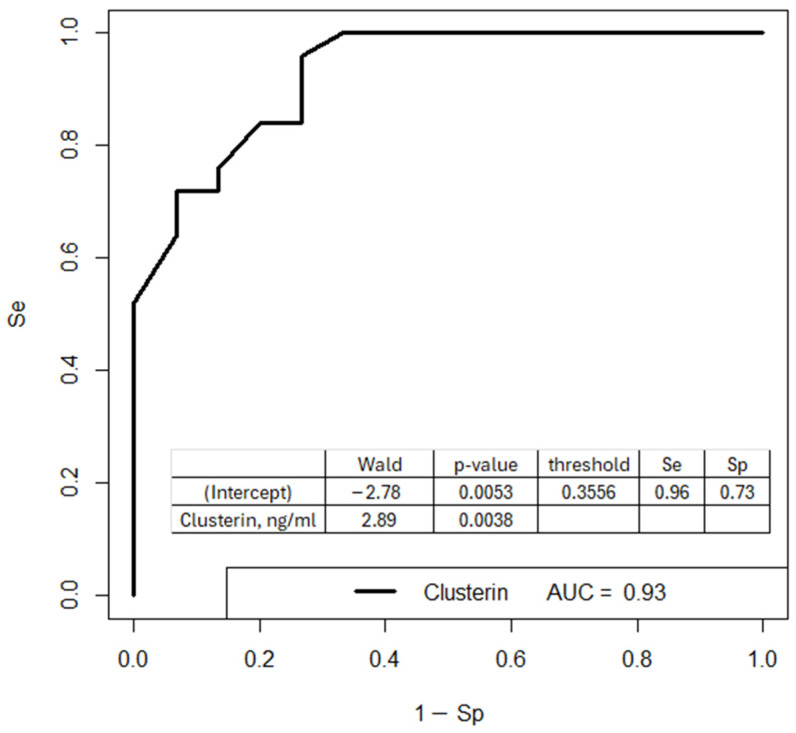
Logistic regression models for diagnosing pregnancy complications (PE or PAS) in the first trimester based on clusterin levels in the vesicular fraction of maternal blood serum at 11–14 weeks of pregnancy.

To implement clusterin analysis via ELISA into clinical practice, a logistic regression model was developed (Figure 6). The dependent variable (outcome) was the diagnosis at delivery: 0, normal pregnancy, and 1, pregnancy complications (PE or PAS). The model parameters are provided in the lower inset of Figure 6.

This model, based on clusterin levels in the vesicular fraction of maternal blood serum, demonstrates 96% sensitivity in diagnosing the development of PE or PAS in pregnant women during the first trimester.

### 3.3. Identifying Correlations Between piRNAs and Protein Molecules Associated with PE or PAS

**Figure 7:**
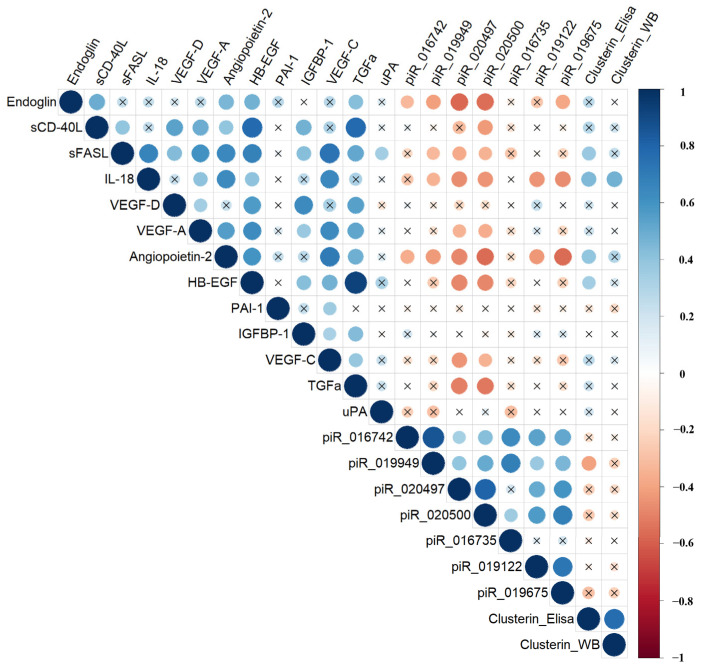
Correlation matrix based on the non-parametric Spearman rank correlation method. Significant (*p* < 0.05) correlations are indicated by a dot, non-significant correlations are indicated by a cross, positive correlations are marked in blue, and negative correlations in red—the more significant the correlation, the larger the dot size.

Using the nonparametric Spearman rank correlation method to assess the strength and significance of relationships between quantitative features, several significant correlations were identified between specific piRNAs and certain proteins in the blood serum of pregnant women at 11–14 weeks of pregnancy (Figure 7, Table S4).

The correlation matrix revealed negative correlations between endoglin concentration and the levels of piR_016742, piR_019949, piR_020497, piR_020500, and piR_019675; sCD-40L and piR_020500; sFASL and piR_019949, piR_020497, and piR_020500; IL-18 and piR_019949, piR_020497, piR_020500, piR_019122, and piR_019675; VEGF-C, HB-EGF, VEGF-A, and TGFα and piR_020497 and piR_020500; angiopoietin and piR_016742, piR_019949, piR_020497, piR_020500, piR_019122, and piR_019675; and clusterin (ELISA data) and piR_019949.

To understand these relationships, potential target genes of the piRNAs that correlate with the analyzed proteins were identified using the miRanda algorithm as described in our recent study [26]. However, none of the identified target genes overlapped with those of the analyzed proteins. Therefore, the observed negative correlations between piRNAs and proteins (Endoglin, sCD-40L, IL-18, VEGF-C, HB-EGF, VEGF-A, TGFα, angiopoietin, and clusterin) may suggest an indirect relationship, possibly via interactions between piRNAs and the mRNAs of transcription factors that regulate the expression of these proteins. Using GeneCard database and Venn diagrams, potential transcription factor targets were identified. Out of 526 transcription factors, 7 transcription factor genes were found to be the potential targets of hsa_piR_019122, hsa_piR_020497, and hsa_piR_019949 (Tables S5 and Table 4). The identified negative correlations between piR_019675 and Endoglin, IL-18, and Angiopoietin-2 might be explained by the direct regulatory effect of piR_019675 on the potential target gene EIF3A (Table S5), which is essential for several steps in protein synthesis initiation.

## 4. Discussion

This study represents the first attempt to create an algorithm for first-trimester pregnancy screening to diagnose placental abnormalities in the cases of PE and PAS using circulating piRNAs and their potential protein targets. The choice of piRNAs as potential biomarker molecules was made due to the abundance of this class of small noncoding RNAs, in contrast to microRNAs (miRNAs), and the higher likelihood of identifying unique molecules for diagnosing pregnancy complications such as PE and PAS before their clinical manifestations. In our previous studies, we identified miRNA biomarkers for detecting these complications during the first trimester of pregnancy [19,20]. However, the lists of these molecules did not overlap, and therefore, no common biomarkers for both PE and PAS were identified.

Using deep sequencing and quantitative real-time PCR, we identified unique piRNA biomarkers for placental anomalies in cases of PE or PAS during first-trimester pregnancy screening. Statistically significant negative correlations were found between piRNAs associated with PE and PAS (hsa_piR_019122, hsa_piR_020497, hsa_piR_019949, and piR_019675) and the quantitatively measured circulating blood proteins involved in EMT, angiogenesis, inflammation, cell proliferation, cell adhesion, and apoptosis, specifically, Endoglin, IL-18, VEGF-C, Angiopoietin-2, sFASL, VEGF-A, HB-EGF, TGFα, and Clusterin. Among the potential target genes identified for these piRNAs, we found the transcription factors GMEB2, SP1, ZEB2, ZNF155, ZNF747, JUNB, and ZFP64 and the translation initiation factor EIF3A, which regulate the expression levels of the circulating proteins mentioned above. Since the role of these proteins in the pathogenesis of PE and/or PAS is well documented [31,32,33,34,35,36,37,38,39,40,41], the presence of statistically significant correlations with these piRNA markers proves their functional significance as diagnostic molecules. Due to the absence of significant differences between the PAS and Norm groups by these proteins unlike the PE group, which significantly differed from Norm group by the level of Angiopoietin-2, IL-18, VEGF-C, sFASL, and Endoglin, it was not possible to develop a unified algorithm for detecting these pregnancy complications in the first trimester. Attempts by other researchers to diagnose PE or PAS in the first trimester of pregnancy using circulating placental factors (VEGF-A, PAPP-A, inhibin A, activin A, P-selectin, interleukin 2, sFlt-1, and PLGF) were also unsuccessful since in the case of PE they are useful for the early onset but not late onset form of the syndrome and in the second and third trimesters but not in the first trimester [42,43,44], and in the case of PAS only, associations with the levels of the circulating proteins were identified without proof of their specificity for PAS and the ability to distinguish from other pregnancy complications [45,46,47,48].

For the first time, we developed an algorithm for first-trimester pregnancy screening based on logistic regression models constructed from the quantitative assessment of the secretory form of clusterin by ELISA and piRNAs (hsa_piR_020497, hsa_piR_019949, piR_019675, and hsa_piR_019122) by quantitative real-time PCR. Based on the clusterin level in the vesicular fraction of maternal blood serum, with 96% sensitivity, it is possible to detect cases of complicated pregnancies (presence of PE or PAS), which can be confirmed by analyzing the levels of hsa_piR_019949 and hsa_piR_020497 in the total serum fraction with 96% sensitivity. To identify one of the complications, the model based on the RT-PCR data for hsa_piR_019675 levels in maternal blood serum can distinguish PE from PAS with 100% specificity. In cases where PE or PAS is not detected using the above models, we recommend confirming the diagnosis using the model based on the quantitative data for hsa_piR_019122, which diagnoses the absence of PE or PAS with 92% specificity.

## 5. Conclusions

The present study is the first to identify universal piwiRNAs (hsa_piR_019122, hsa_piR_020497, hsa_piR_019949, and piR_019675) associated with the development of PE and PAS but differentiating the two pregnancy complications from each other in the first trimester. A significant inverse relationship was found between the level of these piwiRNAs and proteins participating in EMT, angiogenesis, inflammation, cell proliferation, cell adhesion, and apoptosis (endoglin, IL-18, VEGF-C, angiopoietin-2, sFASL, VEGF-A, HB-EGF, TGFα, and clusterin)—processes involved in the invasion of trophoblast cells into the decidual endometrium and placentation, which are impaired in the case of PE or PAS. Based on the quantitative analysis of certain combinations of these piwiRNAs and the secretory form of clusterin in serum plasma of pregnant women, an algorithm for conducting first-trimester screening for early detection of PE or PAS and, as a result, timely interventions to reduce maternal and perinatal morbidity and mortality is proposed. The main limitation of the study is the small cohort of 41 patients included in the study. Obtaining data concerning the performance of the use of this algorithm in a large cohort appears to be critical for implementation into routine clinical practice.

## Figures and Tables

**Table 1:** Parameters of logistic regression models presented in Figure 2.

Model	Wald	*p*-Value	Coefficients	Threshold	Sensitivity	Specificity
*PE vs. Norm*						
*Models from Figure 2A*						
1 Model				0.46	0.83	0.79
(Intercept)	2.01	0.04	0.93			
piR_016742	−2.26	0.02	-1.3			
piR_016735	2.36	0.02	1.51			
4 Model				0.27	1	0.58
(Intercept)	1.68	0.1	1.44			
piR_019949	−2.06	0.04	−0.89			
piR_016735	2.22	0.03	1.19			
13 Model				0.6	0.42	1
(Intercept)	1.99	0.05	4.14			
piR_019122	2.08	0.04	1.43			
*PAS vs. Norm*						
*Models from Figure 2B*						
5 Model				0.36	1	0.77
(Intercept)	2.28	0.02	13.99			
piR_019122	2.25	0.02	4.4			
11 Model				0.25	1	0.69
(Intercept)	2.35	0.02	10.44			
piR_019675	2.35	0.02	2.15			
*(PAS + PE) vs. Norm*						
*Models from Figure 2C*						
1 Model				0.79	0.83	1
(Intercept)	2.33	0.02	2.98			
piR_016742	−2.37	0.02	−2.39			
piR_016735	2.45	0.01	2.93			
3 Model				0.73	0.79	0.92
(Intercept)	2.45	0.01	3.8			
piR_019949	−2.39	0.02	−1.64			
piR_016735	2.47	0.01	2.28			
8 Model				0.52	0.96	0.85
(Intercept)	2.76	0.01	5.65			
piR_019949	−2.23	0.03	−0.79			
piR_020497	2.86	0	1.6			
9 Model				0.68	0.83	0.92
(Intercept)	3.21	0	8.49			
piR_019122	3.11	0	2.49			
10 Model				0.61	0.83	0.85
(Intercept)	2.55	0.01	4.73			
piR_016742	−2.05	0.04	−0.76			
piR_020497	2.89	0	1.49			
15 Model				0.72	0.71	0.92
(Intercept)	2.75	0.01	1.68			
piR_016735	2.83	0	0.74			
17 Model				0.53	0.92	0.77
(Intercept)	2.95	0	5.48			
piR_020497	2.79	0.01	1.35			
18 Model				0.68	0.63	1
(Intercept)	2.64	0.01	3.48			
piR_019675	2.54	0.01	0.65			
*PAS vs. PE*						
*Models from Figure 2D*						
2 Model				0.76	0.83	1
(Intercept)	2.32	0.02	2.44			
piR_019675	2.8	0.01	0.87			
5 Model				0.47	0.83	0.83
(Intercept)	2.31	0.02	7.29			
piR_019122	2.35	0.02	2.99			

**Table 2:** Data from quantitative analysis of proteins in the blood serum of women at 11–14 weeks of pregnancy using the Bio-Plex Pro human cancer biomarker assay, along with comparisons of the corresponding groups using the Wilcoxon–Mann–Whitney U test.

Group	Me(Q1;Q3), pg/mL	*p*-Value		Group	Me(Q1;Q3), pg/mL	*p*-Value	
Angiopoietin-2		PAS	PE	VEGF-A		PAS	PE
PAS	10.48 (9.36; 10.77)	1	0.004	PAS	6.34 (5.95; 6.45)	1	0.12
PE	11.59 (10.85; 11.91)	0.0037	1	PE	6.49 (6.34; 6.74)	0.12	1
Norm	10.38 (9.53; 11.12)	0.84	0.006	Norm	6.15 (5.84; 6.57)	0.61	0.06
Endoglin		PAS	PE	VEGF-C		PAS	PE
PAS	9.14 (8.86; 9.38)	1	0.21	PAS	6.71 (5.75; 6.94)	1	0.01
PE	9.37 (9.01; 9.63)	0.21	1	PE	7.09 (6.84; 7.56)	0.01	1
Norm	8.79 (8.4; 9.27)	0.24	0.02	Norm	6.56 (5.75; 7.02)	0.85	0.01
HB-EGF		PAS	PE	VEGF-D		PAS	PE
PAS	7.31 (6.92; 7.43)	1	0.2	PAS	7.87 (7.58; 8.04)	1	0.28
PE	7.42 (7.26; 7.54)	0.2	1	PE	7.8 (7.52; 7.9)	0.28	1
Norm	7.09 (6.7; 7.54)	0.6	0.08	Norm	7.67 (7.31; 7.88)	0.16	0.65
IGFBP-1		PAS	PE	sCD-40L		PAS	PE
PAS	14.77 (13.99; 15.61)	1	1	PAS	7.67 (7.41; 7.98)	1	0.1
PE	14.88 (14.2; 15.47)	1	1	PE	8.25 (7.97; 8.37)	0.1	1
Norm	15.37 (14.31; 15.84)	0.41	0.37	Norm	7.73 (7.23; 8.06)	0.92	0.07
IL-18		PAS	PE	sFASL		PAS	PE
PAS	1.43 (0.95; 2.03)	1	0.005	PAS	5.25 (4.8; 5.34)	1	0.05
PE	3.5 (2.37; 3.77)	0.005	1	PE	5.44 (5.32; 5.6)	0.05	1
Norm	1.27 (−0.56; 2.45)	0.42	<0.001	Norm	5.25 (4.75; 5.45)	0.94	0.04
PAI-1		PAS	PE	uPA		PAS	PE
PAS	12.56 (11.92; 12.75)	1	0.3	PAS	3.35 (2.83; 4.04)	1	0.07
PE	12.65 (12.36; 13.03)	0.3	1	PE	4.27 (3.35; 4.78)	0.07	1
Norm	12.61 (12.04; 12.92)	0.46	0.64	Norm	3.81 (2.98; 4.69)	0.36	0.35
TGFa		PAS	PE				
PAS	4.78 (4.48; 5.07)	1	0.38				
PE	4.96 (4.52; 5.26)	0.38	1				
Norm	4.71 (4.28; 5.22)	0.66	0.18				

**Table 3:** Comparative analysis of clusterin levels in the vesicular fraction of blood serum in the Norm, PE, and PAS groups.

Group	Me (Q1; Q3)	*p*-Value	
Clusterin, Western Blot		PAS	PE
PAS	0.89 (0.8; 0.97)	1	0.041
PE	1.17 (0.83; 1.27)	0.041	1
Norm	0.72 (0.63; 0.82)	0.01	<0.001
Clusterin, ELISA		PAS	PE
PAS	3.14 (2.76; 3.93)	1	0.597
PE	4.01 (2.28; 5.33)	0.597	1
Norm	1.66 (1; 2.02)	<0.001	<0.001

**Table 4:** Transcription factors that are potential target genes of piRNAs and regulate the expression of genes encoding proteins circulating in peripheral blood during the first trimester of pregnancy.

Analyzed Protein	Transcription Factor for Analyzed Protein *	piRNAs
VEGF-C; VEGF-A; IL18; Clusterin; sFASL; Angiopoietin-2	GMEB2	hsa_piR_019122
Endoglin; sCD-40L; sFASL; IL18; VEGF-A; Angiopoietin-2; HB-EGF; VEGF-C; TGFa; Clusterin	SP1	
Endoglin; sCD-40L; sFASL; IL18; VEGF-A; Angiopoietin-2; HB-EGF; VEGF-C; TGFa; Clusterin	ZEB2	
VEGF-A; Angiopoietin-2	ZNF155	
VEGF-A; Angiopoietin-2	ZNF747	
Endoglin; sCD-40L; sFASL; IL18; VEGF-A; Angiopoietin-2; HB-EGF; VEGF-C;TGFa; Clusterin	JUNB	hsa_piR_020497
Endoglin; VEGF-A; TGFa; Clusterin	ZFP64	hsa_piR_019949

* Target genes of the piRNAs listed in the table. Proteins that significantly negatively correlate with the piRNAs listed in the table are highlighted in red.

## Data Availability

The data presented in this study are available in this article and Supplementary Materials.

## Supplementary Materials

The following supporting information can be downloaded at https://www.mdpi.com/article/10.3390/biom15020228/s1: Table S1: Clinical characteristics of the third cohort of patients. Table S2: Deep sequencing data on piRNA. Table S3: The parameters of logistic regression models based on RT-PCR data on the piRNA level. Table S4: The parameters of the correlation matrix based on the non-parametric Spearman rank correlation method. Table S5: Potential gene targets of piRNAs. File S1: Original Western blot images.
