# Supplementary figures and images for: Universal First-Trimester Screening Biomarkers for Diagnosis of Preeclampsia and Placenta Accreta Spectrum
Source: Biomolecules. 2025 Feb 4;15(2):228. doi: 10.3390/biom15020228 (PMC11852485; doi:10.3390/biom15020228)

The WB original image in Figure 5

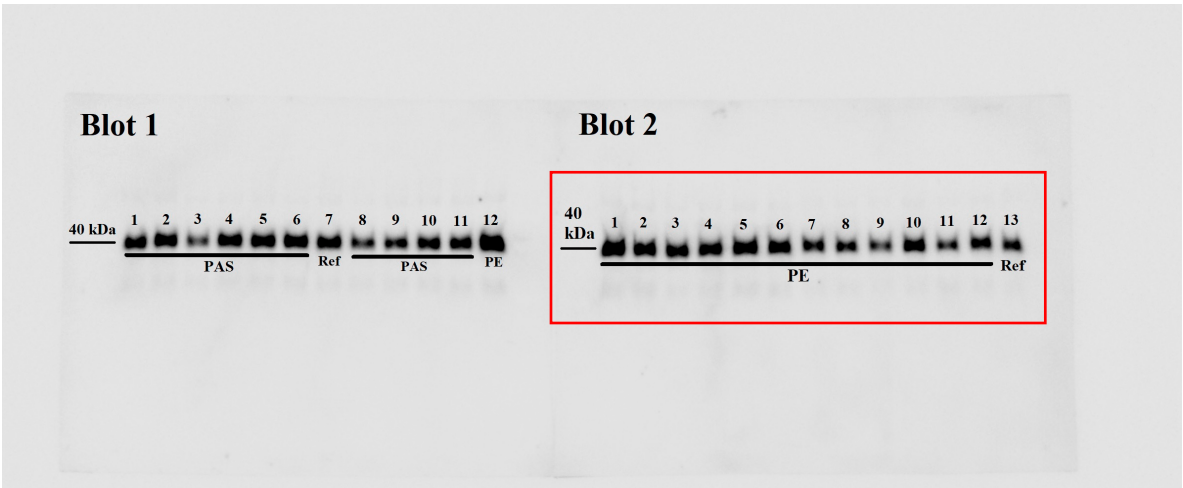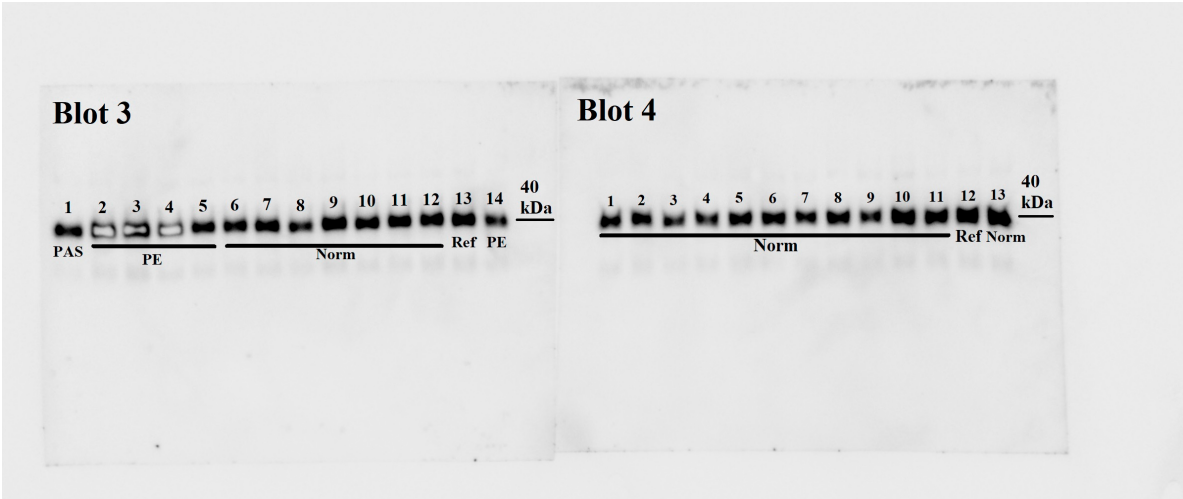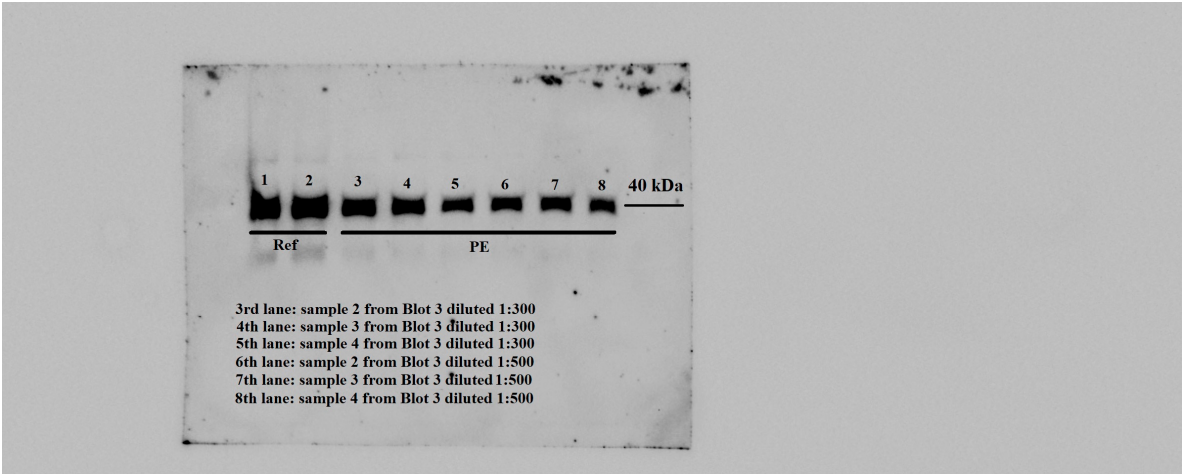

Supplement: Supplementary file 1 [file biomolecules-15-00228-s001.zip › File S1.pdf]
